# Human *Lactococcus garvieae* Bloodstream Infection Complicated by Spondylodiscitis, Germany

**DOI:** 10.3201/eid3208.260334

**Published:** 2026-08

**Authors:** Philipp Schulz, Pascal Migaud, Daniela Drauz, Sebastian Jaeger, Johannes Elias, Hartmut Stocker

**Affiliations:** Joseph-Kliniken–St. Joseph Krankenhaus Berlin–Tempelhof, Berlin, Germany (P. Schulz, P. Migaud, D. Drauz, S. Jaeger, H. Stocker); Charité–Universitätsmedizin Berlin, Berlin (P. Schulz, P. Migaud, H. Stocker); MDI Limbach Berlin GmbH, Berlin (J. Elias); Health and Medical University Potsdam, Potsdam, Germany (J. Elias)

**Keywords:** Lactococcus garvieae, zoonoses, bacteria, streptococcaceae, lactococcus, endocarditis, spondylitis, chemical and drug induced liver injury, case reports, Germany

## Abstract

*Lactococcus garvieae* bloodstream infection in a woman in Germany resulted in spondylodiscitis and bioprosthetic mitral valve endocarditis. Six weeks of ceftriaxone followed by 10 days of doxycycline led to sustained clinical and microbiological resolution. This case highlights the need for thorough diagnostic testing and individualized, shared decision-making in infective endocarditis cases.

*Lactococcus garvieae* bacteria causes hemorrhagic septicemia in fish (*1*). Human infections are rare (41 reported cases; Appendix 1) and presumably foodborne; they primarily manifest as infective endocarditis (IE) (*2*). We report *L. garvieae* bloodstream infection and IE in a woman in Germany.

In January 2025, we admitted an 85-year-old woman for conservative management of spondylodiscitis. She reported a 4-month history of progressive back pain, lower extremity edema, and night sweats; she underwent bioprosthetic mitral valve (PMV) implantation 4 years earlier for severe mitral regurgitation (Appendix 2 Table 1). Transthoracic echocardiography 15 months prior was unremarkable. She reported daily smoked fish consumption.

Physical examination revealed a holosystolic heart murmur best heard at the apex, pitting lower limb edema, and an Osler node. Laboratory investigations showed elevated leukocytes (14,580 cells/µL [reference range 3,990–10,490 cells/µL]), C-reactive protein (19.8 mg/dL [reference <0.5 mg/dL]), and procalcitonin (0.14 ng/mL [reference <0.06 ng/mL]).

We withheld empirical antimicrobial therapy until pathogen identification. *L. garvieae* bacteria was isolated from 4 blood cultures collected during the first 2 hospitalization days and confirmed by MALDI-TOF (matrix-assisted laser desorption ionization time-of-flight) mass spectrometry. Using published susceptibility patterns (*3*), we initiated intravenous ampicillin (2 g every 6 h) on hospitalization day 3, then switched to intravenous ceftriaxone (2 g every 12 h) the next day.

Transesophageal echocardiography (TEE) demonstrated 6 × 16 mm vegetations on both PMV leaflets (Figure 1, panel A). Comprehensive imaging revealed no additional embolic lesions. Urinalysis was unremarkable, but rheumatoid factor was positive. Because the patient met 2 major (microbiological, imaging) and 2 minor (immunologic, predisposing PMV) published IE criteria (*4*), we diagnosed PMV-IE.

**Figure 1:**
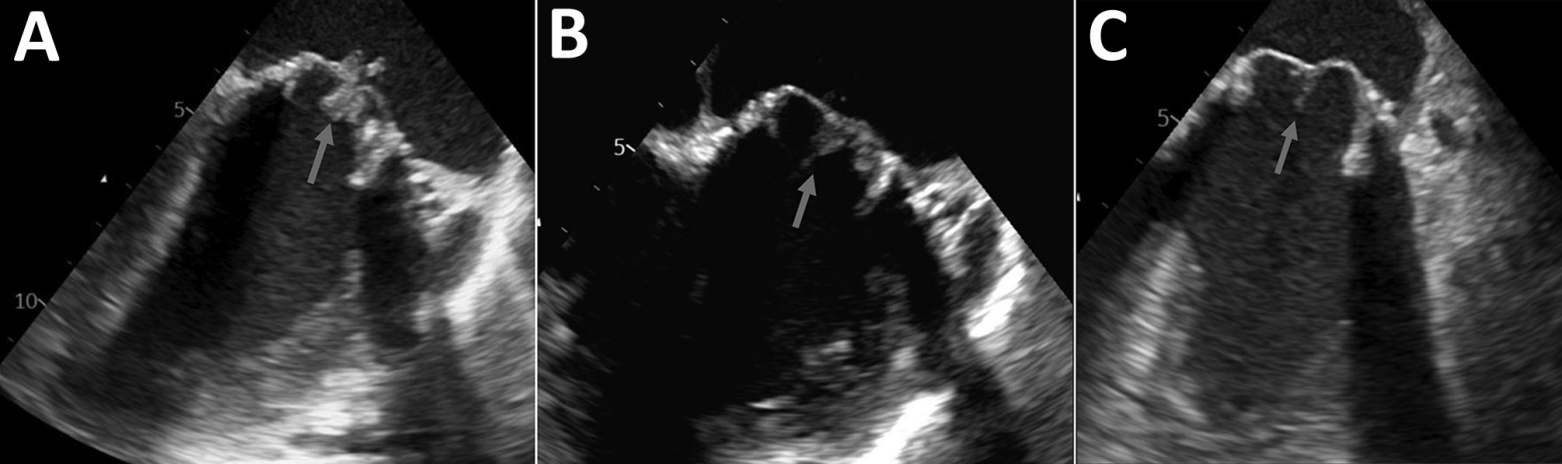
Transesophageal echocardiograph findings from a case of human *Lactococcus garvieae* bloodstream infection complicated by spondylodiscitis, Germany. A) Echocardiograph obtained on day 6 demonstrates mobile vegetations on both leaflets of the biological prosthetic mitral valve with a maximum dimension of 6 × 16 mm and pronounced valvular leaflet thickening (arrow). No notable valvular dysfunction was present. B) Echocardiograph obtained on day 45 shows marked regression of mitral valve leaflet thickening and a smaller vegetation (arrow; maximum 4 × 4 mm). No notable valvular dysfunction was present. C) Echocardiograph obtained on day 98 shows marked regression of mitral valve leaflet thickening. Arrow shows a faint 5 mm filamentous structure that might represent a residual finding. No notable valvular dysfunction was present.

Our endocarditis team reviewed the patient’s case. European Society of Cardiology guidelines recommend surgery within 3–5 days for vegetations >10 mm because of increased risk for embolism or death (*5*). However, benefits of surgery solely on the basis of vegetation size remain uncertain, and embolic risk decreases after starting antimicrobial therapy. Our patient’s estimated French Embolic Risk Score was 6% at week 1, 9% at week 2, and 11% at week 26, but EuroSCORE II indicated a 12% perioperative risk for death. Because embolic risk was low but perioperative risk for severe injury or death remained high, the patient, in consultation with the medical teams, chose to continue conservative management with ceftriaxone for 6 weeks.

Repeat blood cultures remained sterile; inflammatory markers declined (Figure 2). TEE on day 16 demonstrated a smaller (4 × 16 mm) but more mobile vegetation. The patient’s remaining hospitalization was unremarkable. She was discharged on 4 weeks of oral doxycycline (100 mg 2×/d) for a residual 4 × 4 mm vegetation (Figure 1, panel B) and elevated C-reactive protein (6 mg/dL).

**Figure 2:**
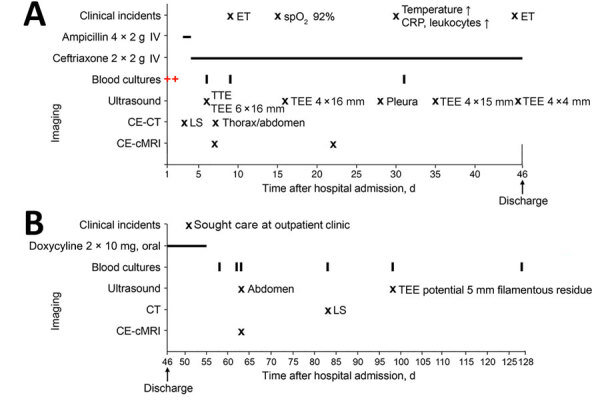
Summary of the clinical course from a case of human *Lactococcus garvieae* bloodstream infection complicated by spondylodiscitis, Germany. A) Clinical course during hospitalization, days 1–46. B) Clinical course and follow-up after initial discharge, days 46–128. Red plus signs indicate collection of blood, cultures of which were positive for *L. garvieae*; black vertical lines indicated negative blood cultures. CE-CT, contrast-enhanced computed tomography; CE-cMRI, contrast-enhanced cerebral magnetic resonance imaging; CRP, C-reactive protein; ET, endocarditis team consulted; IV, intravenous; LS, lumbar spine; spO_2_, peripheral oxygen saturation; TEE, transesophageal echocardiograph; TTE, transthoracic echocardiograph.

On postdischarge day 6, the patient sought care in our outpatient clinic for new-onset persistent nausea and mixed hepatocellular and cholestatic liver injury. We suspected idiosyncratic drug-induced liver injury because of rapid onset after doxycycline initiation and a Roussel Uclaf Causality Assessment Method score of 8 (probable drug-induced liver injury). HIV and hepatitis A–C and E tests and an abdominal ultrasound were unremarkable. After discontinuing doxycycline, the patient’s symptoms and laboratory values improved (Appendix 2 Figure). Follow-up at day 98 showed no new abnormalities and near-complete resolution of PMV vegetation (Figure 1, panel C). The patient remained clinically stable 11 months after discharge (Figure 2, panel B).

*L. garvieae* was initially described as *Streptococcus garvieae* in 1983 but reclassified in 1985 (*2*). Biochemical tests can misidentify *L. garvieae* as *Streptococcus* or *Enterococcus* spp. bacteria (*6*); therefore, diagnosis relies on MALDI-TOF mass spectrometry or ribosomal sequencing. IE is the most common manifestation of human *L. garvieae* infection (*2*), and 61% (22/36) of cases involved fever; patients sought medical attention a median of 15.5 (IQR 6.75–30) days after symptom onset (Appendix 1; Appendix 2 Table 2). The subacute, afebrile course in this case is unusual, but up to 55% of spondylodiscitis and 20% of IE case-patients are afebrile (*7*,*8*); thus, absence of fever does not rule out either diagnosis. Of note, the 2023 European Society of Cardiology update incorporated spondylodiscitis as a minor diagnostic criterion for IE (*5*), but the modified Duke criteria did not (*4*).

Genetic similarity between clinical and gastrointestinal *L. garvieae* isolates supports foodborne transmission and opportunistic pathogenicity (*2*). Infection likely is enabled by underlying gastrointestinal pathology, as reported in 67% (24/36) of *L. garvieae* endocarditis cases (Appendix 2 Table 2). The isolate from this case showed broad antimicrobial resistance, comparable to those from other human infections (Appendix 2 Tables 2–4). One aquaculture study showed 83% of tested *L. garvieae* strains harbored ≥2 resistance genes (*9*). Thus, from a One Health perspective, *L. garvieae* could serve as a gut reservoir for transferable antimicrobial resistance genes and opportunistic infections (*2*,*10*).

In summary, this rare case of multidrug-resistant zoonotic *L. garvieae* bloodstream infection illustrates the value of comprehensive diagnostic testing for spondylodiscitis and withholding antibiotics until pathogen identification. This case also highlights the complexity of IE management; clinical context requires interdisciplinary, individualized strategies transcending guidelines. Transparent communication of risks and uncertainties is essential for evidence-informed, patient-centered care.

Appendix 1 Detailed cases of human *Lactococcus garvieae* infection used to investigate *L. garvieae* bloodstream infection complicated by spondylodiscitis, Germany.

Appendix 2 Additional information on human *Lactococcus garvieae* bloodstream infection complicated by spondylodiscitis, Germany.
