# Supplementary material for: Human Lactococcus garvieae Bloodstream Infection Complicated by Spondylodiscitis, Germany
Source: Emerg Infect Dis. 2026 Aug;32(8):1363–5. doi: 10.3201/eid3208.260334 (PMC13426888; doi:10.3201/eid3208.260334)
Supplement: Appendix 2 — Additional information on human Lactococcus garvieae bloodstream infection complicated by spondylodiscitis, Germany. [file 26-0334-Techapp-s2.pdf]

Article DOI: https://doi.org/10.3201/eid3208.260334

EID cannot ensure accessibility for Appendix materials supplied by authors. Readers who have difficulty accessing Appendix content should contact the authors for assistance.

Human *Lactococcus garvieae* Bloodstream Infection Complicated by Spondylodiscitis, Germany

Appendix 2

Appendix 2 Table 1. Medications at Admission and Past Medical History

Medication	Morning	Noon	Evening	Night	Indication
Acetylsalicylic Acid, mg	100	0	0	0	Bioprosthetic mitral valve
Ramipril, mg	2.5	0	2.5	0	Arterial hypertension
Torasemide, mg	2.5	0	0	0	Peripheral edema (congestive heart failure)
Rosuvastatin, mg	0	0	0	5	Dyslipidemia
Pantoprazole, mg	0	0	40	0	Acetylsalicylic Acid
Hydromorphone, mg	2	0	2	0	Back pain (spinal canal stenosis Th2-3, L3-5)
Metamizole, g	1	1	1	1	Back pain (spinal canal stenosis)
Venlafaxine, mg	150	0	75	0	Depression
Polyethylene Glycol, g	52.5	52.5	52.5	0	Irritable Bowel Syndrome, credominant
Bioprosthetic mitral valve					Mitral regurgitation (severe)

*Epic E100-29M, Abbott Laboratories, https://www.abbott.com.

Appendix 2 Table 2. Summary of Clinical, Microbiological, and Therapeutic Characteristics of All 36 Reported *Lactococcus garvieae* Cases with Available Data

Parameter	Value
Baseline characteristics	
Median age, y (IQR)	69 (59.5–78.25)
Sex	
M	22 (61.1)
F	14 (38.9)
Comorbidities	
Arterial Hypertension	13 (36.1)
Atrial Fibrillation	10 (27.8)
Diabetes Mellitus Type 2	7 (19.4)
Dyslipidemia	6 (16.7)
Coronary Artery Disease	6 (16.7)
Congestive Heart Failure	4 (11.1)
Risk Factors	
GI pathology, any	24 (66.7)
Gastrointestinal polyps	8 (22.2)
Diverticula	5 (13.9)
Gastritis	4 (11.1)
Peptic ulcer disease	3 (8.3)
Fish Exposure	16 (44.4)
Ingestion	14 (87.5)
Handling	2 (12.5)
No Fish Exposure	9 (25.0)
Not reported	11 (30.6)
Outcome	
Alive	32 (88.8)
Antimicrobial + Surgical Therapy	8 (75.0)
Antimicrobial Therapy only	24 (25.0)
Monotherapy	7 (29.2)
Combination Therapy	17 (70.8)

Parameter	Value
Embolic Complication during therapy	3 (9.4)
No Embolic Complication during therapy	29 (90.6)
Dead	4 (11.1)
Antimicrobial + Surgical Therapy	2 (50.0)
Antimicrobial Therapy only	2 (50.0)
Heart Valve	
Number of Affected Valves	
Mono-valvular	30 (83.3)
Multi-valvular	6 (16.7)
Valve Location	
Aortic valve, n = 42	21 (50.0)
Native, n = 21	8 (38.1)
Prosthetic, n = 21	13 (61.9)
Mitral valve, n = 42	20 (41.7)
Native, n = 20	15 (75.0)
Prosthetic, n = 20	5 (25.0)
Tricuspid valve, n = 42	1 (2.4)
prosthetic	1 (100)
Valve Type	
Native, n = 45	23 (51.1)
Prosthetic, n = 45	22 (48.9)
Biological, n = 22	13 (59.1)
Mechanical, n = 22	5 (22.7)
not reported, n = 22	4 (18.2)
Clinical Presentation	
Duration of Presenting Complaint, median d (IQR)	15.5 (6.75– 30)
Most Common Presenting Complaints	
Fever	22 (61.1)
Pain	13 (36.1)
Muscle	4 (30.8)
Back	3 (23.1)
Head	3 (23.1)
Joint	2 (15.4)
Chest	1 (7.7)
Abdominal	1 (7.7)
Anorexia	8 (22.2)
Malaise	7 (19.4)
Chills	6 (16.7)
Dyspnea	6 (16.7)
Weakness	5 (13.9)
Weight loss	5 (13.9)
Laboratory Investigations on Admission, median (IQR)	
Hemoglobin, g/dL; n = 14	9.9 (9.0–10.5)
Leucocytes, cells/µL; n = 25	12,735 (9,250–15,500)
C-reactive Protein, mg/dL; n = 22	9.1 (5.40–21.33)
Creatinine, mg/dL; n = 10	1.41 (1.1–1.56)
Microbiology methods used	
Biochemical	15 (41.7)
VITEK 2†	9 (25.0)
API Strep (20 or 32)	6 (16.7)
Rapid ID 32 Strep	4 (66.7)
API 20 Strep	1 (16.7)
Not reported	1 (16.7)
BD Phoenix System‡	1 (6.7)
16s rRNA/DNA-sequencing	14 (38.9)
MALDI-TOF	13 (36.1)
Not reported	9 (25.0)
Combination of Microbiological Methods for Pathogen Identification	
Biochemical	3 (8.3)
API Strep	1 (33.3)
VITEK†	1 (33.3)
Not reported	1 (33.3)
+ 16s rDNA/rRNA sequencing	10 (27.8)
+ 16s rDNA/rRNA sequencing + MALDI-TOF	2 (5.6)
MALDI-TOF	7 (19.4)
+ 16s rDNA/rRNA sequencing	4 (11.1)
Polymerase Chain Reaction	1 (2.8)
Not reported	9 (25.0)
Antimicrobial Therapy	

Parameter	Value
Antibiotic regimen	
Combination therapy	26 (72.2)
Monotherapy	10 (27.8)
Median therapy duration among 32 surviving patients, d (IQR)	42 (42–42)
<42 days	5 (15.6)
42 days	18 (56.3)
>42 days	6 (18.8)
Not reported	3 (9.4)
Most Common Antimicrobials Used	
Gentamicin	28 (77.8)
Ceftriaxone	22 (61.1)
Aminopenicillin (Amoxicillin or Ampicillin)	18 (50.0)
+ beta-Lactamase-Inhibitor	4 (11.1)
Vancomycin	12 (33.3)
Penicillin	9 (25.0)
Levofloxacin	4 (11.1)
Antimicrobial Resistance, n = 24	
Penicillin§	6 (25.0)
Gentamicin	4 (16.7)
Penicillin	1 (4.2)
Ampicillin	2 (8.3)
Ampicillin/Sulbactam	1 (4.2)
Piperacillin/Tazobactam	1 (4.2)
Rifampin	1 (4.2)
Norfloxacin	1 (4.2)
Bacitracin	1 (4.2)

*Values are no. (%) except as indicated. MALDI-TOF, matrix-assisted laser desorption ionization time-of-flight.
†bioMérieux, https://www.biomerieux.com.
‡Becton, Dickinson and Company, https://www.bd.com.
§When used as monotherapy in infective endocarditis according to clinical breakpoints issued by the European Committee on Antimicrobial Susceptibility Testing (EUCAST) for viridans group streptococci.

Appendix 2 Table 3. Results of Antimicrobial Susceptibility Testing

Medication	Disk diffusion	E-test (MIC, mg/L)
Penicillin	Resistant	Resistant (1)
Ampicillin	Resistant	ND
Ampicillin/Sulbactam	Resistant	ND
Piperacillin/Tazobactam	Resistant	ND
Cefuroxim	Resistant	ND
Cefotaxim	Susceptible	ND
Ceftriaxone	Susceptible	Susceptible (0.38)
Vancomycin	Susceptible	ND
Clindamycin	Resistant	ND
Tetracycline	ND	Susceptible (0.38)
Levofloxacin	ND	Intermediate (0.75)†
Gentamicin	ND	Resistant (1.5)

*The isolate from blood cultures on day 2 underwent disk-diffusion susceptibility testing; by this method, minimal inhibitory concentrations (MICs) are not generated. Following the initial disk-diffusion testing, the stored isolate was retested later using an E-test, enabling determination of MICs. Susceptibility results are based on breakpoints from alpha-hemolyzing *Streptococcus spp.* for penicillin (using the endocarditis-specific breakpoint), ceftriaxone and gentamicin (Screen), as well as *Staphylococcus spp.* for tetracycline and levofloxacin. ND, not done.
†Susceptible at increased exposure.

Appendix 2 Table 4. Antimicrobial Resistance Profile of Lactococcus garvieae Isolates Reported in the Literature

Author (reference no.)	Year	Country	Breakpoint from	Susceptibility (MIC, mg/L)		
				Resistant	Intermediate	Susceptible
Fleming (*1*)	2012	USA (acquired in South Korea)	CLSI recommendations for viridian streptococci	Clindamycin, Ampicillin, Gentamicin	/	Vancomycin, Ampicillin/Sulbactam, Ceftriaxone, Piperacillin/Tazobactam
Russo (*2*)	2012	Italy	EUCAST (2026; v 16.0) breakpoints for Viridans group streptococci	Clindamycin (>64), Rifampin (>64), Bacitracin (>64), Norfloxacin, Penicillin* (2)		Erythromycin (0.125), Cefotaxime (0.5), Levofloxacin (0.5), Tetracycline (0.5), Imipenem (0.023), Ampicillin (0.25), Amoxicillin/Clavulanic Acid (0.5), Ciprofloxacin (0.75), Daptomycin (0.125), Vancomycin (2), Gentamicin (2), Teicoplanin (0.5)
Vinh (*3*)	2006	Canada	CLSI criteria for *Streptococcus spp*. other than *Streptococcus pneumoniae*	Clindamycin	Penicillin	Ampicillin, Vancomycin
			CLSI criteria for *Enterococcus spp.*	Clindamycin, Norfloxacin	Erythromycin, Nitrofurantoin	Penicillin G, Chloramphenicol, Ciprofloxacin, Ofloxacin, Levofloxacin, Tetracycline, and Vancomycin, Gentamicin, Streptomycin
Kitagawa (*4*)	2024	Japan	Not specified	Gentamicin	/	Penicillin, Ampicillin, Cefazolin, Cefmetazole, Imipenem, Minocycline, Vancomycin
Cabrales (*5*)	2020	Colombia	Not specified	Gentamicin	/	Ampicillin, Ceftriaxone, Ciprofloxacine
Clavero (*6*)	2017	Chile	CLSI (2015) for β-hemolytic streptococci	Clindamycin, Penicillin*	/	Vancomycin, Cefotaxime
Fihman (*7*)	2006	France	EUCAST (2026; v 16.0) breakpoints for Viridans group streptococci	Penicillin* (0.75)	/	Amoxicillin (0.5), Cefotaxime (0.38), Vancomycin (1.5), Teicoplanin (0.38)
Lim (*8*)	2017	England	EUCAST (2026; v 16.0) breakpoints for Viridans group streptococci	Penicillin* (1)		Gentamicin
Lim (*9*)	2018	Singapore	EUCAST (2026; v 16.0) breakpoints for Viridans group streptococci	Penicillin* (0.75)		Ceftriaxone (0.25), Gentamicin (3.0)
Rasmussen (*10*)	2014	Sweden	EUCAST (2026; v 16.0) breakpoints for Viridans group streptococci	Penicillin* (0.5)		Tobramycin (2)
Watanabe (*11*)	2011	Japan	EUCAST (2026; v 16.0) breakpoints for Viridans group streptococci	Clindamycin (>256), Penicillin* (0.5)		Ceftriaxone (0.38), Erythromycin (0.25), Vancomycin (0.38), Linezolid (2)

*When used as monotherapy in infective endocarditis according to clinical breakpoints issued by the European Committee on Antimicrobial Susceptibility Testing (EUCAST) for Viridans group streptococci.

References

1. Fleming H, Fowler SV, Nguyen L, Hofinger DM. *Lactococcus garvieae* multi-valve infective endocarditis in a traveler returning from South Korea. Travel Med Infect Dis. 2012;10:101–4. PubMed https://doi.org/10.1016/j.tmaid.2012.02.004

2. Russo G, Iannetta M, D'Abramo A, Mascellino MT, Pantosti A, Erario L, et al. Lactococcus garvieae endocarditis in a patient with colonic diverticulosis: first case report in Italy and review of the literature. New Microbiol. 2012;35:495–501. PubMed

3. Vinh DC, Nichol KA, Rand F, Embil JM. Native-valve bacterial endocarditis caused by Lactococcus garvieae. Diagn Microbiol Infect Dis. 2006;56:91–4. PubMed https://doi.org/10.1016/j.diagmicrobio.2006.02.010

4. Kitagawa I, Ishikawa N, Ono R. Infective endocarditis caused by *Lactococcus garvieae*: a case report and review of the literature. IDCases. 2024;36:e01941. PubMed https://doi.org/10.1016/j.idcr.2024.e01941

5. Cabrales HJ, García-Posada MJ, Porto-Valiente JM, Espinosa A, Narváez Y. Bacteremia due to *Lactococcus garvieae*: first case in Colombia [in Spanish]. Infectio. 2020;24:193. https://doi.org/10.22354/in.v24i3.865

6. Clavero R, Escobar J, Ramos-Avasola S, Merello L, Álvarez F. *Lactococcus garvieae* endocarditis in a patient undergoing chronic hemodialysis. First case report in Chile and review of the literature [in Spanish]. Rev Chilena Infectol. 2017;34:397–403. PubMed https://doi.org/10.4067/s0716-10182017000400397

7. Fihman V, Raskine L, Barrou Z, Kiffel C, Riahi J, Berçot B, et al. *Lactococcus garvieae* endocarditis: identification by 16S rRNA and *sodA* sequence analysis. J Infect. 2006;52:e3–6. PubMed https://doi.org/10.1016/j.jinf.2005.04.021

8. Lim FH, Jenkins DR. Native valve endocarditis caused by *Lactococcus garvieae*: an emerging human pathogen. BMJ Case Rep. 2017;2017:bcr2017220116. PubMed https://doi.org/10.1136/bcr-2017-220116

9. Lim SM, Wong B, Cross GB, Merchant R. *Lactobacillus garvieae* endocarditis presenting with leg cramps. IDCases. 2018;13:e00427. PubMed https://doi.org/10.1016/j.idcr.2018.e00427

10. Rasmussen M, Björk Werner J, Dolk M, Christensson B. *Lactococcus garvieae* endocarditis presenting with subdural haematoma. BMC Cardiovasc Disord. 2014;14:13. PubMed https://doi.org/10.1186/1471-2261-14-13

11. Watanabe Y, Naito T, Kikuchi K, Amari Y, Uehara Y, Isonuma H, et al. Infective endocarditis with *Lactococcus garvieae* in Japan: a case report. J Med Case Rep. 2011;5:356. PubMed https://doi.org/10.1186/1752-1947-5-356

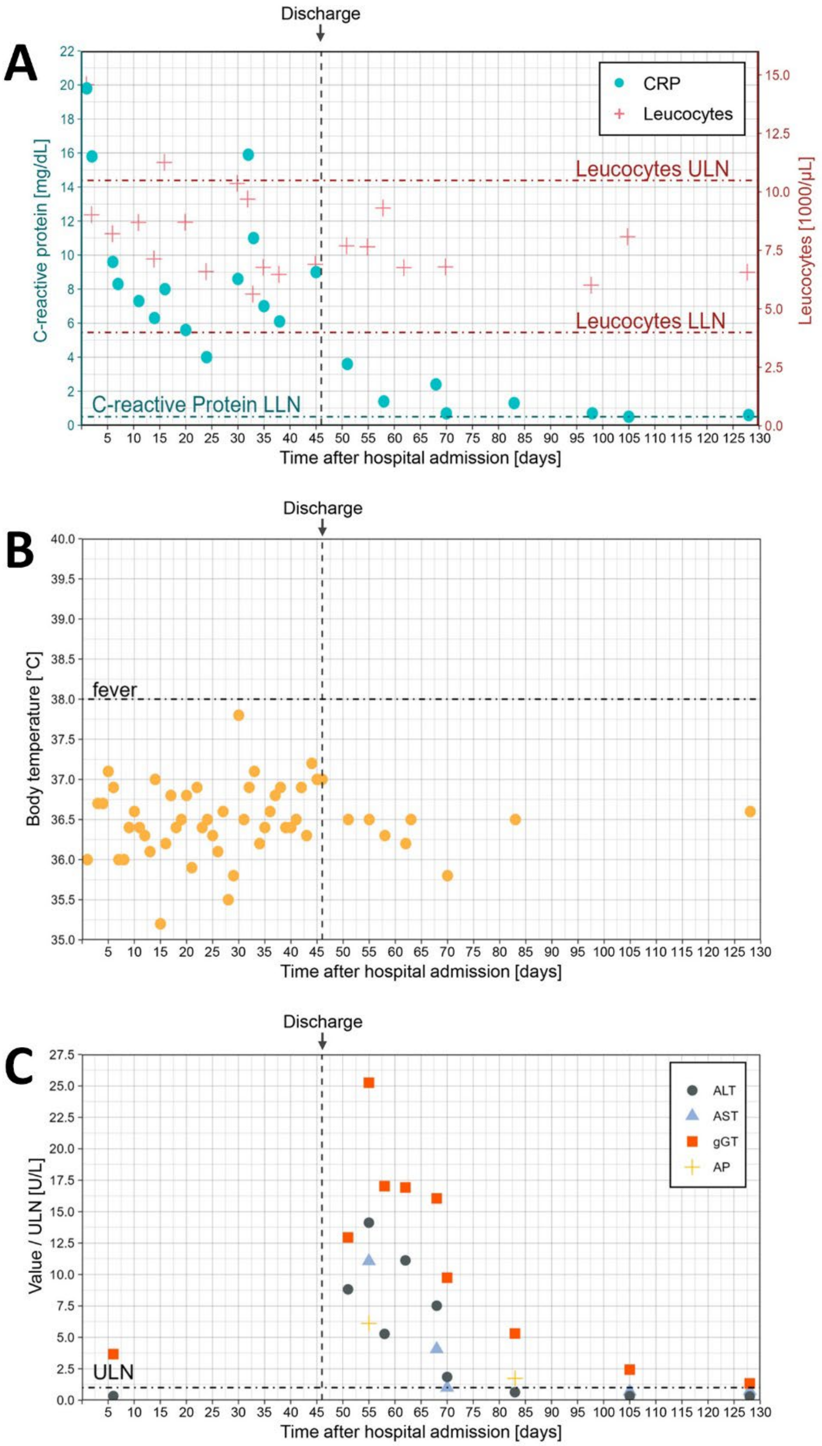

Appendix 2 Figure*.* Temporal Trends in Laboratory Biomarkers and Body Temperature. A) C-reactive Protein and Leucocyte Count. B) Body temperature, measured using a digital ear thermometer. C) Liver enzymes, displayed relative to their respective upper limit of normal. ALT, alanine aminotransferase; AST, aspartate aminotransferase; AP, alkaline phosphatase; CRP, C-reactive protein; gGT, gamma-glutamyl transferase; LLN, lower limit of normal; ULN, upper limit of normal.
